# Task-shifting or problem-shifting? How lay counselling is redefining mental healthcare

**DOI:** 10.1371/journal.pmen.0000067

**Published:** 2024-06-17

**Authors:** Liana Chase, Parbati Shrestha, Gaurav Datta, Nicky Forsythe, Sumeet Jain, Sujen Man Maharjan, Kaaren Mathias, Xandra Miguel-Lorenzo, Shubha Ranganathan, Sujan Shrestha, Kripa Sidgel, Prasansa Subba, Kamal Gautam, Dristy Gurung, Maura Cranny Ntow

**Affiliations:** 1 Department of Anthropology, Durham University, Durham, United Kingdom; 2 Transcultural Psychosocial Organization-Nepal, Kathmandu, Nepal; 3 Department of Biomedical Sciences, University of North Dakota, Grand Forks, North Dakota, United States of America; 4 Talk for Health, London, United Kingdom; 5 School of Social and Political Science, University of Edinburgh, Edinburgh, United Kingdom; 6 Faculty of Health, University of Canterbury, Christchurch, New Zealand; 7 Department of Applied Health Research, UCL, London, United Kingdom; 8 Department of Anthropology, LSE, London, United Kingdom; 9 Department of Liberal Arts, Indian Institute of Technology Hyderabad, Sangareddy, Telangana, India; 10 Department of Global Health and Social Medicine, Kings College London, London, United Kingdom; 11 Department of Psychology, Tribhuvan University, Kathmandu, Nepal; 12 Possible Nepal, Kathmandu, Nepal; 13 Department of Primary Care and Mental Health, University of Liverpool, Liverpool, United Kingdom; 14 Department of Health Services and Population Research, Kings College London, London, United Kingdom; 15 Department of English, Germanic and Romance Studies, University of Copenhagen, Copenhagen, Denmark; PLOS: Public Library of Science, UNITED KINGDOM

We are in the midst of a sea change in the way talk therapy is delivered globally. Recent years have seen unprecedented support for lay counselling, or the delivery of talk-based therapeutic interventions by people who do not hold a clinical degree. In low-resource settings, practitioners in the field of global mental health have generated a substantial body of evidence suggesting lay counsellors can play a role in addressing care gaps [1, 2]. In high-resource settings, lay counselling responds to inequitable access to talking therapies and a movement to involve people with lived experience in care delivery [3, 4].

We write as a group of scholars and practitioners concerned with the way lay counselling is represented in the global mental health literature. Our experiences are varied: we have engaged with lay counselling in urban clinics, remote and underserved communities, religious congregations, and humanitarian emergencies. Yet we have come to share a common conviction–that mental healthcare is being fundamentally redefined through the involvement of lay providers.

To fully grasp this transformation requires looking beyond technical narratives that predominate in the field of global health. In existing literature, lay counselling is primarily described in terms of ‘task-shifting’, or the delegation of clinical responsibilities to less trained providers [2]. In this representation, lay counsellors are conceptualized as a ‘delivery mechanism’–a quick and cost-effective way of extending the reach of formal mental health services [5].

Contemporary work in the social sciences challenges this simplistic representation of lay counsellors. Leading social theories of disease argue that the contours of medicine’s objects depend to a large extent upon the practices through which we seek to engage them [6]. In this view, terms like ‘mental illness’ never simply describe natural entities in the world, for the techniques used to render illnesses ‘visible, audible, tangible, knowable’ inevitably influence how we draw their boundaries [6]. It follows that transformations in clinical practice–such as the adaptation of therapies for lay delivery–will inevitably alter how we come to know and understand the suffering they seek to address.

From this theoretical perspective, the rise of lay counselling globally involves not only the shifting of tasks, but also shifts in the very definition of a ‘mental health problem’. Most significantly, lay counsellors lack the skills and supervision needed to formulate distress in terms of universal diagnostic frameworks; instead, their care is generally oriented toward lay expressions of distress reflecting local cultural and moral norms. In rural Nepal, for example, counselling clients may be told they are suffering from a ‘wound in the heart-mind’ [7]. In the Solomon Islands, Christian counselling practices described locally as ‘disentangling’ or ‘straightening out’ take as their object women’s broken social relations with family members and God [8]. While lay counsellors in some regions of India have been mobilized to enforce medicine compliance, other innovative programmes support counsellors to address social problems such as isolation [9, 10]. And in the UK, an emerging cadre of peer counsellors operate on the assumption that, rather than illness, they are addressing deficits of connection and community in modern society, making their interventions more analogous to vitamins than to medicines [11].

Lay counselling thus entails much more than a logistical reconfiguration of service delivery. Through the rise of lay counselling globally, the remit of ‘mental healthcare’ is rapidly expanding to encompass a wide range of everyday expressions of distress and dysfunction. These changes in the way distress comes to be recognized and labelled within therapeutic encounters have far-reaching implications; they affect the subjectivity and life trajectories of people using services, the responses of their families and communities, and wider public perceptions of mental health and illness.

We see an urgent need to move beyond technical representations of lay counselling as ‘task-shifting’ to develop new metaphors that better accommodate social theory and lived experience. Thinking in more nuanced ways about lay counselling is essential not only to comprehend the wider effects of this shift in practice, but also to harness its transformative potential for global mental health. Rather than striving to fit lay counselors into existing frameworks, consideration should be given to how lay counsellors can address problems long associated with conventional mental health services, including rigid clinical hierarchies, excessive medicalization, and deficit-based understandings of mental health. Moving beyond a fixation on improving lay counsellors’ fidelity to existing models, we propose asking how we can build on the knowledge, assets, and resources these community members already possess [12]. Instead of treating lay counselling as a means of replicating and reinforcing biomedical expertise, we might approach it as an entirely different and indispensable form of expertise in itself–the kind of expertise that is not one-sided but allows shared roles and responsibilities in the healing and recovery process. Such a shift would contribute to ensuring the service user is more than just a ‘recipient’ of care.

Reconceptualizing lay counselling also has implications for research. The generation of evidence on lay counselling has been dominated by intervention trials measuring symptom reduction, which tell us little about how and why the practice works or its wider consequences. Research on ‘key ingredients’ in the field of psychotherapy suggests the importance of the relationship between therapist and client, but has largely neglected the influence of different relational norms and values in non-Western contexts [13]. We see an urgent need for theoretically grounded and cross-cultural research on lay counselling in the social sciences. Open-ended, qualitative methodologies such as ethnography are needed to understand what mental ill health *is* for people engaging with lay counselling in diverse social contexts and to generate contextualized insights into the novel opportunities and dilemmas the approach presents (including emerging risks such as ‘task-dumping’ and perpetuating stigma [14]). Participatory and coproduced studies are moreover essential to ensure the voices of lay counsellors and people with lived experience shape future directions in the field [15]. In short, we call for an inclusive lay counselling research agenda rooted in the awareness that new therapeutic practices do not merely respond to the world; they help to shape it.
